# Epstein–Barr Virus–Associated Encephalitis in an Immunocompetent Adult: A Case Report From Thailand

**DOI:** 10.1155/crdi/8336608

**Published:** 2025-09-22

**Authors:** Ekachai Singhatiraj, Pavis Laengvejkall, Put Chaikamnerd, Krit Pongpirul

**Affiliations:** ^1^Department of Internal Medicine, Bumrungrad International Hospital, Bangkok, Thailand; ^2^King Mongkut's Institute of Technology Ladkrabang, Bangkok, Thailand; ^3^Clinical Research Center, Bumrungrad International Hospital, Bangkok, Thailand; ^4^Center of Excellence in Preventive and Integrative Medicine, Faculty of Medicine, Chulalongkorn University, Bangkok, Thailand

## Abstract

We report a case of Epstein–Barr virus (EBV) encephalitis in a 27-year-old man from Bangkok, Thailand, presenting with fever and altered mental status. Cerebrospinal fluid analysis showed neutrophil predominance and EBV-positive PCR. The patient improved with intravenous dexamethasone, highlighting EBV as a potential encephalitis pathogen in immunocompetent individuals.

## 1. Introduction

Epstein–Barr virus (EBV) is a herpesvirus (Human herpes virus 4), widely disseminated through intimate contact between susceptible individuals and asymptomatic carriers. Most primary EBV infections worldwide are subclinical. EBV antibodies are present in approximately 95% of adults globally [1]. While primary infection is usually asymptomatic in childhood, it can induce infectious mononucleosis (IM) during adolescence or adulthood [2]. EBV is generally transmitted through oral secretions. Though rare, EBV can also cause central nervous system (CNS) involvement, such as demyelinating disease, encephalitis, meningitis, cranial or peripheral nerve palsies, and acute cerebellar ataxia [3, 4].

A 27-year-old man with a history of childhood epilepsy was admitted to an outside hospital with a 1-day fever and myalgia, followed by altered mental status. On examination, he was drowsy but had no focal neurological signs. Initial laboratory results showed lymphocytosis with atypical lymphocytes and elevated liver enzymes (Table 1). Suspecting a rickettsial infection, the patient was started on doxycycline 100 mg BID.

**Figure 1:**
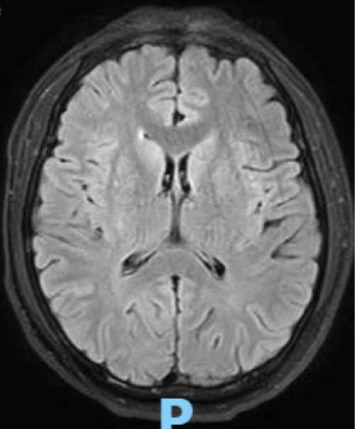
MRI finding shows normal parenchyma and ventricles, with no evidence of extra-axial fluid collection, mass lesion, or mass effect.

On Day 3, the patient's condition deteriorated, and he became less responsive with neck stiffness. Lumbar puncture revealed an opening pressure of 22 cmH_2_O, cloudy fluid, elevated protein (97 mg/dL), glucose of 55 mg/dL, and 215 white blood cells/mm^3^ (99% polymorphonuclear cells). Intravenous meropenem and azithromycin were administered to cover bacterial and rickettsial meningitis. Brain MRI was unremarkable (Figure 1), and EEG showed diffuse slowing without seizure activity.

On Day 7, his neurological condition had not improved, so dexamethasone 4 mg IV q 6 h was started, and the patient was transferred to our hospital for further care. On arrival, he remained drowsy without focal neurological signs. Repeat cerebrospinal fluid (CSF) analysis showed 60 WBCs/mm^3^ (85% lymphocytes), protein of 74.8 mg/dL, and glucose of 82 mg/dL. A CSF encephalitis panel revealed positive EBV by PCR. EEG demonstrated mild to moderate diffuse cerebral dysfunction without epileptiform discharges. He was diagnosed with viral meningoencephalitis due to EBV. Intravenous antibiotics were discontinued, but steroid therapy was continued with a tapering dosing with dexamethasone 4 mg PO q 12 h on Day 8 and discontinued on Day 10. The patient's condition improved since then, becoming more alert and able to follow simple commands. Upon discharge, he was able to speak slowly but appropriately. At his 2-week follow-up, he had fully recovered both clinically and on laboratory evaluation.

Encephalitis is an inflammation of the brain parenchyma, characterized by neurological dysfunction and evidence of CNS inflammation through CSF pleocytosis or findings on neuroimaging/EEG. EBV is implicated in approximately 6% of viral encephalitis cases in children [5]. EBV encephalitis typically presents with neurological symptoms without preceding infectious mononucleosis. A review of EBV encephalitis cases shows that most patients present with fever, headache (83%), lymphocytic pleocytosis, moderate hyperproteinorachia, and a positive EBV PCR test in 87% of cases, with a mortality rate of 15.5% [6]. EBV infection of the CNS can be classified into two categories, which include CNS diseases associated with primary or reactivated EBV infections, and CNS syndromes related to chronic EBV infections. It can manifest concurrently with IM during an acute phase of the disease, or present lately during a convalescent phase of illness [4]. The pathogenesis of EBV encephalitis is still unclear. Neurological complications usually occur concurrently with typical manifestations of infectious mononucleosis; however, they may also present during the resolution phase of infection. The possible mechanisms are described as direct viral invasion to brain parenchyma, the infiltration of cytotoxic T-lymphocytes into the neural tissue, and antibody–antigen complex deposition in neural structures [7]. Postmortem studies in specific cases have identified infiltrated lymphocytes containing EBV DNA in meninges and perivascular areas, with their absence in neurons indicating inflammatory brain damage resulting from an immune response rather than direct viral invasion, in contrast to herpes simplex virus (HSV) infection [8, 9]. We believe that the main pathogenetic cause of EBV-associated CNS infection is the result of the inflammatory response by the body's immune system rather than direct viral invasion, so systemic corticosteroids are more likely to be efficacious. In our case, the patient presented with fever, headache, and altered mental status. Initial CSF analysis showed neutrophil predominance, which later shifted to lymphocytes. Serology confirmed acute EBV infection with positive IgM, IgA, and IgG antibodies to EBV-CA. He received steroids on Day 7, and his neurological has improved gradually since then. He came to follow up 1 month after discharge from the hospital with a full recovery.

Currently, there is no established treatment for EBV encephalitis. Although acyclovir has in vitro activity against the lytic phase of EBV infection [10], clinical data do not support its use in treating acute IM [11]. Steroid therapy may be helpful in severe cases, especially those complicated by airway obstruction, liver failure, or aplastic anemia; whereas the effectiveness of corticosteroids in HSV encephalitis is not proven [12, 13]. In this case, antiviral agents were not given due to the lack of proven efficacy of acyclovir and the delayed presentation of symptoms (Day 7). Steroid treatment resulted in gradual improvement. It is believed that immune-mediated mechanisms rather than direct viral invasion are the primary cause of EBV-associated CNS infection. Further research is needed to explore therapeutic interventions for this condition.

## Figures and Tables

**Table 1:** Laboratory data.

	Normal ranges	Day 0	Day 1	Day 3	Day 4	Day 7
*Blood*						
WBC count	4.5–11.0 × 10^3^/μL	6.58	5	14.2		5.6
Neutrophils	37%–80%	21	28	34		39
Lymphocytes	10%–50%	67	67	61		49.3
Atypical lymphocytes	0%	9	2	4		
Monocytes	2%–9%	2	2	1		9
Eosinophils	0%–4%	1	1			1.8
Hemoglobin	14.0–18.0 g/dL	14.5	14.9	13.6		14.5
Hematocrit	42.0%–52.0%	43.9	44	42.7		42.7
Platelets	140–450 × 10^3^/μL	171	159	145		312
Glucose	65–115 mg/dL					130
BUN	8–21 mg/dL					13.3
Creatinine	0.8–1.5 mg/dL					0.82
AST (SGOT)	17–40 units/L		284	247		191
ALT (SGPT)	21–72 units/L		484	426		398
EBV viral load	0 copies/mL					243

*Cerebrospinal fluid*						
Opening pressure	6–25 cmH_2_O			22		
WBC	0–5			230	150	60
Neutrophils	0%–6%			99	64	15
Lymphocytes	40%–80%			1	36	85
Protein	12–40 mg/dL			97	83	74.8
Sugar	40–70 mg/dL			55	66	82
